# A three-arm randomised controlled trial comparing Gonadotrophin Releasing Hormone (GnRH) agonist long regimen *versus *GnRH agonist short regimen *versus *GnRH antagonist regimen in women with a history of poor ovarian response undergoing in vitro fertilisation (IVF) treatment: Poor responders intervention trial (PRINT)

**DOI:** 10.1186/1742-4755-4-12

**Published:** 2007-12-28

**Authors:** Sesh K Sunkara, Arri Coomarasamy, Yakoub Khalaf, Peter Braude

**Affiliations:** 1 Assisted Conception Unit, Guy's Hospital, Guy's and St Thomas' Hospitals NHS Foundation Trust, London, SE1 9RT, UK; 2 Dept of Women's Health, 10^th ^Floor, North Wing, St Thomas' Hospital, Guy's and St Thomas' Hospitals NHS Foundation Trust, London, SE1 7EH, UK

## Abstract

**Background:**

Poor response to ovarian stimulation with exogenous gonadotrophins occurs in 9–24% of women undergoing in vitro fertilisation (IVF) treatment, which represents an estimated 4000–10,000 women per year in the UK. Poor responders often have their treatment cycle cancelled because of expected poor outcome.

One treatment strategy that may influence outcome is the choice of pituitary suppression regimen prior to the initiation of ovarian stimulation. The three commonly used pituitary suppression regimens in IVF treatment are:

(1) the GnRH agonist *long *regimen,

(2) the GnRH agonist *short *regimen and

(3) the GnRH *antagonist *regimen.

A systematic review of randomised controlled trials of these pituitary suppression regimens has shown the evidence to be either inconclusive or inconsistent. We therefore designed a three arm randomised trial to evaluate the effectiveness of these regimens in women who had poor ovarian response in a previous IVF treatment cycle.

**Methods/design:**

Consenting, eligible women will be randomised to one of the three regimens using an internet-based trial management programme that ensures allocation concealment and employs block randomisation and minimisation for prognostic variables. The primary outcome is the number of oocytes retrieved. Other outcomes include total dose of follicle stimulating hormone (FSH) used for ovarian stimulation, *mature *oocytes retrieved, embryos available for transfer, implantation rate and clinical pregnancy rate.

The sample size for this trial has been estimated as 102 participants with 34 participants in each of the three arms. Appropriate interim analysis will be conducted by a Data Monitoring and Ethics Committee (DMEC), and the final analysis will be by intention to treat.

**Trial registration:**

ISRCTN27044628

## Background

Poor ovarian response, defined as failure of the development of sufficient number of mature follicles to proceed to oocyte retrieval or yielding only a few oocytes following gonadotrophin stimulation in women undergoing in vitro fertilisation (IVF) treatment, occurs in 9–24% of women [1]. Poor ovarian response is likely to be an increasing problem with women delaying childbearing and presenting for treatment later in their reproductive life. In comparison to normal responders these patients have impaired fertilisation rates, lower embryo quality and decreased pregnancy rates [2]. Poor responders often have their treatment cycle cancelled because of expected poor outcome [3]. This causes emotional distress for the couple, as well as a financial burden on the couple or the service provider.

Treatment of poor responders who are undergoing IVF treatment remains a challenge. Various treatment regimens and interventions have been proposed in an effort to improve ovarian response and IVF outcome in this group of patients. These include different regimens for pituitary suppression, controlled ovarian hyperstimulation (COH) as well as adjuvant therapies [4]. Currently, the choice of pituitary suppression regimens proposed for the management of poor responders is mainly based on individual centre's or clinician's preferences, with no single protocol considered to be superior over the other. Of the various available pituitary suppression regimens, the three commonly used ones are:

(1) the GnRH agonist long regimen,

(2) the GnRH agonist short regimen and

(3) the GnRH antagonist regimen.

**Figure 1:**
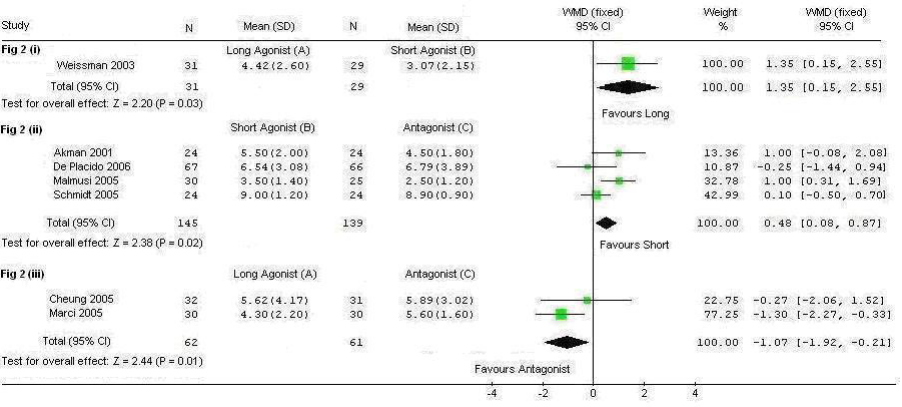
Meta-analysis of the number of oocytes retrieved with different pituitary suppression regimens in poor responders.

To address the question of their effectiveness, we performed a systematic review and meta-analysis. Literature searches were conducted in MEDLINE, EMBASE, the Cochrane Database, National Research Register and ISI proceedings to identify randomised trials comparing the effects of the above three regimens. We identified seven randomised controlled trials comparing the regimens with each other.[5-11] There were no trials that compared all three regimens against each other. The quality of these 7 randomised trials was generally poor (only 2/7 trials had adequate allocation concealment reported). These trials were also generally small, leading to imprecision in their findings, even when combined in a meta-analysis (Figure 1). Furthermore, there was substantial clinical and statistical heterogeneity amongst the trials. The findings were inconclusive or inconsistent (Figure 1). A well designed, adequately powered, three arm trial comparing the long GnRH agonist regime versus the short GnRH agonist regime versus the GnRH antagonist regime is therefore needed.

## Methods/design

### Objective

In the proposed trial we will evaluate which of the three commonly used down regulation regimens is the most effective for women who have shown poor response in their previous IVF treatment cycle.

### Design

A prospective, allocation concealed, assessor-blind, three-arm randomised-controlled-trial.

### Primary endpoints

• Number of eggs retrieved per IVF treatment cycle started.

### Secondary endpoints

• The total dose of follicle stimulating hormone (FSH) used for ovarian stimulation

• The number of mature eggs retrieved

• The number of embryos available for transfer

• The clinical pregnancy rate and

• The embryo implantation rate

### Inclusion Criteria

Any woman undergoing an IVF treatment cycle who fits the criteria to be a "poor responder" is eligible to participate in the trial.

For this study a "poor responder" is defined as a woman who had a previous IVF treatment cycle in which she was stimulated with a daily dose of FSH of 300 IU or more and

• Produced an inadequate number of mature follicles (three or less follicles measuring > 17 mm) following stimulation for at least nine days; OR

• Had three or less oocytes retrieved at oocyte retrieval.

### Exclusion Criteria

• Women aged over 40 years.

• Women with a single ovary.

### Randomisation

Third party, distant, internet-based block randomisation with minimisation will be used to ensure randomisation and complete allocation concealment. The aim of minimisation would be to balance for the following prognostic variables (age, body mass index, previous pregnancies and previous live birth). Women may be randomised into the study by internet randomisation [12] or by telephoning a central office.

### Blinding

The trial will be a single-blinded study, where the assessors (the doctor performing the egg collection procedure and the embryologist involved in identifying and assessing the eggs) are blinded to the treatment regimen.

### Methods

**Figure 2:**
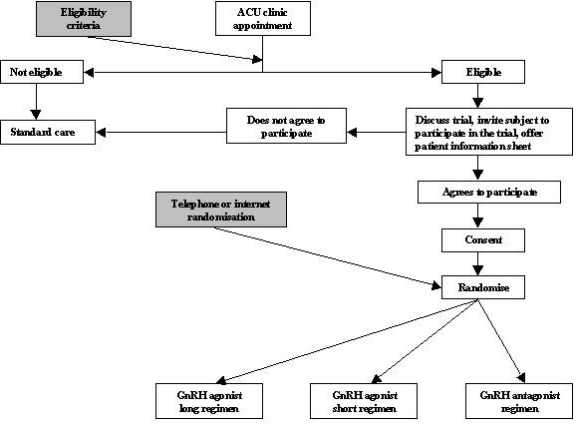
Participant flow chart through the PRINT trial.

All women who have had poor ovarian response in a previous IVF treatment cycle and who wish to undertake another IVF treatment cycle are invited to participate in the trial. The trial details are explained to the patient by a doctor at their clinic appointment. An information sheet [see Additional file 1] explaining the trial is given which also has a contact number to telephone and speak to the trial investigators. If the woman subsequently agrees to participate in the trial she is requested to sign a consent form [see Additional file 2]. The woman is then randomised to one of the three trial regimens and informed which regimen she has been randomised. Should the woman need more time to decide the clinic doctor will gain verbal permission for the investigators to call and ascertain whether the patient wishes to enrol on the trial. If the patient does wish to enrol then one of the investigators will make an appointment for the patient to sign the consent form. If the woman declines to participate then no further action is required with regard to the trial and the woman will receive standard care. The flowchart in figure 2 describes the participant flow in the trial. Details of all participants and outcomes are recorded on the case report form [see Additional file 3].

### The treatment regimens

#### GnRH agonist long regimen (Figure 3)

**Figure 3:**
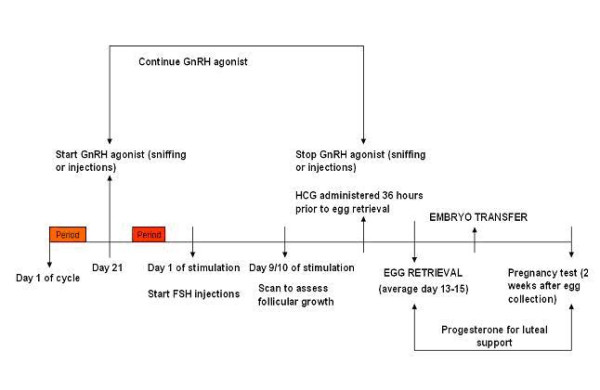
GnRH agonist long regimen.

Pituitary down-regulation with GnRH agonist, nafarelin (Synarel; Pharmacia, UK) starts on day 21 of the menstrual cycle. Nafarelin nasal spray is taken at a dose of two sniffs, twice a day, where one sniff equals 200 micrograms. The woman will generally have menstruation within two weeks of starting nafarelin at which point she attends the assisted conception unit (ACU) for a transvaginal ultrasound scan (TVS) to confirm down regulation (quiescent ovaries with follicles < 10 mm diameter and endometrium < 5 mm in thickness). On confirmation of down regulation, ovarian stimulation is commenced with FSH, follitropin alfa (Gonal-F; Serono, UK) at 450 IU daily by subcutaneous injection. The dose of the nafarelin nasal spray is halved (one sniff, twice a day) during ovarian stimulation. The woman then attends for a TVS nine days after starting ovarian stimulation and is instructed to administer huaman chorionic gonadotrophin (HCG) subcutaneously when criteria for egg collection are met (see below). FSH and nafarelin are continued until HCG administration.

The approximate duration of this treatment regimen (ie from the start of nafarelin nasal spray, commenced on day 21 of the menstrual cycle, until the pregnancy test is performed) is 6 weeks.

#### GnRH agonist short regimen (Figure 4)

**Figure 4:**
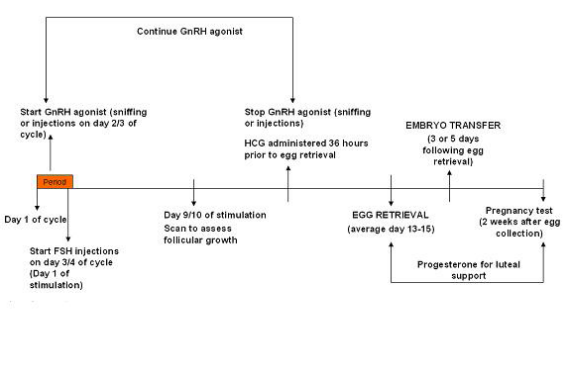
GnRH agonist short regimen.

Treatment for this protocol starts on day 2 or 3 of the menstrual cycle after a TVS has confirmed the ovaries are quiescent and the endometrium thin (<5 mm). The ovaries are generally quiescent and the endometrium thin at the beginning of a menstrual cycle. On the 2nd or 3rd day of the menstrual cycle the woman commences nafarelin nasal spray (one sniff twice a day, where each sniff equals 200 micrograms). This is continued until administration of the HCG injection (approximately 10 to 15 days from the start of nafarelin nasal spray). On the 3rd or 4th day of the menstrual

cycle the woman commences FSH (450 IU daily by subcutaneous injection). The woman attends for a TVS on day 9 of ovarian stimulation and is instructed to administer HCG when criteria for egg collection are met (see below). FSH and nafarelin are continued until the day of HCG administration.

The approximate duration of this treatment regimen (ie from the start of nafarelin nasal spray, commenced on day 2 or 3 of the menstrual cycle, until the pregnancy test is performed) is 4 weeks.

#### GnRH antagonist regimen (Figure 5)

**Figure 5:**
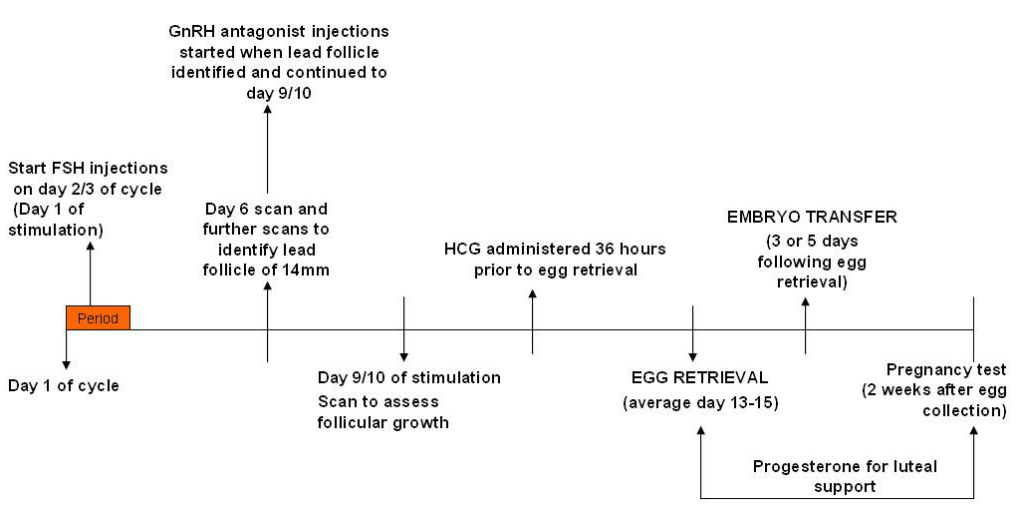
GnRH antagonist regimen.

Treatment with this regimen starts on day 2 or 3 of the menstrual cycle. The woman will attend the ACU for a TVS and if the ovaries are quiescent and the endometrium thin (<5 mm) she will commence ovarian stimulation with a daily dose of 450 IU of FSH subcutaneously. Following this a TVS is performed on day 6 of ovarian stimulation to identify the leading follicle. Scans are continued until the leading follicle has a diameter of 14 mm which is usually between days 6 to day 8 of ovarian stimulation. When the leading follicle has reached a diameter of 14 mm, GnRH antagonist, cetrorelix (Cetrotide; Serono, UK) 0.25 mg daily is administered subcutaneously. The next TVS is performed on day 9 of ovarian stimulation, when the woman is instructed to administer HCG if criteria are met (see below). FSH and cetrorelix are continued until HCG administration.

The approximate duration of this treatment regimen (ie from the start of FSH injections, commenced on day 2 or 3 of the menstrual cycle, until the pregnancy test is performed) is 4 weeks.

The following steps of the treatment are the same for all the three treatment regimens.

### Criteria for HCG administration

When ≥ 3 follicles attain a mean diameter of ≥ 17 mm, HCG, choriogonadotrophin alfa injection (Ovitrelle; Serono, UK) 6,500 IU is administered subcutaneously.

### Egg collection (oocyte retrieval)

Egg collection is performed 34–38 hours after the HCG injection. Egg collection is routinely done under heavy sedation.

### Embryo transfer (replacement of embryos)

Embryo Transfer is performed two, three or five days following the day of egg collection, based on the number and quality of embryos available.

### Luteal support

All women undergoing IVF treatment are advised to self administer progesterone pessaries (Cyclogest; Alpharma, UK) 400 mg daily starting on the day of egg collection until the day of the pregnancy test, then until 8 weeks of pregnancy if the treatment is successful.

### Pregnancy test

All women are requested to do a home urinary pregnancy test 14 days from the day of egg collection.

### Early pregnancy scans

All women having IVF treatment have two early pregnancy scans at 6 and 8 weeks gestation.

### Trial statistics

#### Number of participants

Sample size calculation was based on the observed differences in eggs collected from existing literature as well as on the judgement on what constitutes as a clinically Minimally Important Difference (MID), which was judged to be increasing the number of oocytes retrieved from 3 to 5. For this difference of 2 oocytes retrieved, with a standard deviation of 2.5 (as observed in the existing literature), for a power of 90% and an alpha of 5%, 102 women in total will need to be recruited (34 each of the three arms of the trial).

### Statistical analysis

The analysis will be by intention to treat, and will be carried out in the following four steps:

#### Step 1: Summarising trial data

Baseline data and outcome data will be summarised separately. For continuous variables (eg age and FSH level), we will examine the distribution of the observations, and if normally distributed we will then summarise them as means with standard deviations (SDs). If they are non-normally distributed, then medians and inter-quartile ranges (IRQs) will be reported. For dichotomous data (eg clinical pregnancy), we will provide proportions (or percentages).

For our main outcome measure (the number of oocytes collected), as the number of oocytes is not a dichotomous or continuous variable but is a count, and as it is more likely not to be normally distributed, a model for count such as the negative binomial model will be used for analysis.

In addition to the baseline and outcome data, we will also summarise the recruitment numbers, those participants lost to follow-up, protocol violations and other relevant data.

#### Step 2: Inter-group comparisons

A test for overall comparison (eg ANOVA, or if assumption for ANOVA are not met, a non-parametric equivalence such as Kruskal-Wallis) will be employed for each outcome across all three interventions (A, B, and C), and if this is found to be significant (at a p-value of ≤ 0.05), then we will proceed to pair-wise comparisons (i.e., A vs B, B vs C, A vs C). We appreciate that multitudes of pair-wise comparisons can suffer from Type I (false-positive) error, and will therefore adjust for multiplicity of comparisons (by using steps such as Bonferroni and Tukey's procedure).

The statistical procedures for pair-wise comparisons will depend on the nature of the data: for example, for dichotomous outcomes, we will use Fishers Exact Test or chi-square as appropriate, and for continuous outcomes we will use t-test if the observations in each trial arm are normally distributed; if non-normally distributed, then Mann-Whitney-U test will be employed. Although p-values will be reported, the focus will be on providing 95% confidence intervals around point estimates as these are more useful in interpreting the findings of the trial.

#### Step 3: Sub-group analysis

We will give emphasis to planned (a priori) sub-groups in our analyses. However, we are aware sub-groups analysis can suffer from false positive (due to multiplicity of comparisons) and false negative (due to reduced sample sizes) results, and will place limited importance in subgroup findings in relation to the overall (global) findings. We will use post-hoc subgroup analysis only for the purpose of hypothesis generation.

#### Step 4: Adjustments and sensitivity analyses

If randomisation fails to achieve balanced groups, then we will perform secondary analyses in which we will adjust for unbalanced prognostic factors using procedures such as logistic regression. If the primary unadjusted analysis and secondary adjusted analysis are at discordance, then we will give greater weighting to the primary analysis in the interpretation of trial findings.

For issues such losses to follow-up, missing data, and protocol violations, we will attempt sensitivity analyses to explore the effect of these factors on the trial findings. As a secondary analysis, we will adjust for missing data using imputation techniques to explore the effects of such imputations on the trial findings.

### Interim analysis and data and safety monitoring

The trial will be monitored by a Data Monitoring and Ethics Committee (DMEC). The DMEC has members with clinical and statistical background, who have no conflict of interest relating to the three trial protocols and have no involvement in running of any part of the trial. The DMEC is responsible for Data and Safety Monitoring. During the trial the DMEC will perform interim analysis and review unblinded outcome data for safety and efficacy. The first interim analysis is scheduled to take place after primary outcome data are available for 40% of the trial participants, and subsequently at four monthly intervals.

### Ethics and confidentiality

This trial will be conducted according to the Principles of Good Clinical Practice as defined in the Medicines for Human Use (Clinical Trials) Amended Regulations 2006, the Research Governance Framework for Health & Social Care 2005 and the Data Protection Act. The trial has already received a favourable ethical opinion from a Regional Ethics Committee (REC).

Patient notes containing their personal and treatment details will be kept within the ACU according to the statutory requirements of the HFE Act 1990 and the strict confidentiality that it requires. Notes from patients who have achieved a pregnancy will be kept or archived for 50 years. Patients will need to give their written consent before any of their treatment details or personal information is passed to their General Practitioners or any other persons who are not covered by an HFEA licence.

## Abbreviations

ACU – Assisted Conception Unit

ANOVA – Analysis of variance

COH – Controlled ovarian hyperstimulation

DMEC – Data Monitoring and Ethics Committee

FSH – Follicle stimulating hormone

GnRH – Gonadotrophin releasing hormone

HFEA – Human Fertilisation and Embryology Act

HCG – Human chorionic gonadotrophin

IRQ – Inter-quartile range

ISRCTN – International Standardised Randomised Controlled Trial Number

IU – International Unit

IVF – In vitro fertilisation

MID – Minimally Important Difference

PRINT – Poor responders intervention

REC – Regional Ethics Committee

SD – Standard deviation

TVS – Transvaginal scan

UK – United Kingdom

## Competing interests

The author(s) declare that they have no competing interests.

## Authors' contributions

SKS participated in the design of the study and was involved in drafting the manuscript.

AC participated in the design and development including the statistical analysis plan.

YK participated in drafting the manuscript and revising it critically for important intellectual content.

PB conceived of the study and participated in its design.

## Supplementary Material

Additional file 1 Poor Responders Intervention Trial – Participant information sheet. The data provided represents the information that is given to all women who are eligible to participate in the study. Click here for file

Additional file 2 Poor Responders Intervention Trial – Consent form. The data provided represents the consent form that is signed by eligible women wishing to participate in the trial. Click here for file

Additional file 3 Trial case report form. The data provided represents the form in which data from all participating women are recorded. Click here for file
