# Supplementary material for: A three-arm randomised controlled trial comparing Gonadotrophin Releasing Hormone (GnRH) agonist long regimen versus GnRH agonist short regimen versus GnRH antagonist regimen in women with a history of poor ovarian response undergoing in vitro fertilisation (IVF) treatment: Poor responders intervention trial (PRINT)
Source: Reprod Health. 2007 Dec 28;4:12. doi: 10.1186/1742-4755-4-12 (PMC2259311; doi:10.1186/1742-4755-4-12)
Supplement: Additional file 1 — Poor Responders Intervention Trial – Participant information sheet. The data provided represents the information that is given to all women who are eligible to participate in the study. [file 1742-4755-4-12-S1.doc]

# Poor Responders Intervention Trial

PARTICIPANT INFORMATION SHEET

You are being invited to take part in a research study. Before you make your decision, it is important for you to understand why the research is being done and what it will involve. Please take time to read the following information carefully. You may want to talk to others about the study before taking part.

- Part 1 tells you the purpose of this study and what will happen to you if you take part.
- Part 2 gives you more detailed information about the conduct of the study.

Ask us if there is anything that is not clear or if you would like more information. Take time to decide whether or not you wish to take part.

Part 1

What is the purpose of the study?

There are three commonly used regimens used to suppress the pituitary hormones during In Vitro Fertilisation (IVF). The purpose of this research is to find out which of these is the most effective for women who have shown a poor response in their previous treatment cycle(s). There is currently no evidence to say which gives the best outcome.

It is necessary during IVF treatment to control the reproductive cycle. In order to do this drugs are used to suppress the reproductive hormones released by the pituitary gland in the brain. These hormones are the Follicle Stimulating Hormone (FSH) and the Luteinising Hormone (LH). Both these hormones are stimulated by the Gonadotrophin Releasing Hormone (GnRH).

There are two types of drugs which suppress the pituitary hormones. The first is a GnRH agonist, called Nafarelin. An agonist is a drug which mimics the action of a naturally occurring substance in the body. Nafarelin activates the pituitary just like the GnRH in the body, but while the GnRH triggers the release of hormones by repeated on/off pulses, Nafarelin in IVF treatment delivers a long, sustained burst which keeps the pituitary in the ‘off’ mode.

The second drug is a GnRH antagonist, called Cetrorelix. An antagonist is a drug which opposes the action of a naturally occurring substance in the body. In this way, Cetrorelix prevents the release of pituitary hormones.

There are three different regimes used to block the release of the reproductive hormones. They are as follows:

1. **The GnRH agonist (Nafarelin) long regimen**

- Day 21 of menstrual cycle: start Nafarelin nasal spray (continued until HCG injection)

↓ approx 2 weeks later

- Ovarian stimulation with FSH injections (continued until HCG injection)

↓ approx 9-14 days later

- HCG injection (to mature the eggs)

↓ 36 hours later

- Egg collection *

↓ 2, 3, or 5 days later

- Embryos put back into the womb

↓ 2 weeks later

- Pregnancy test
- *Starting on the day of egg collection, you will take a daily dose of Progesterone (to prepare the lining of the womb), which is taken in the form of pessaries inserted into the vagina. These should be taken daily up until the pregnancy test. If the test is positive, the pessaries should be continued until the 8^th^ week of pregnancy.
- *The approximate duration of this treatment regimen (from the start of Nafarelin*

*until the pregnancy test is performed) is 6 weeks.*

**2. The GnRH agonist (Nafarelin) short regimen**

- Day 2/3 of menstrual cycle: Start Nafarelin nasal spray (continued until HCG injection)
- Day 3/4: Ovarian stimulation with FSH injections (continued until HCG injection)

↓ approx 9-14 days later

- HCG injection (to mature the eggs)

↓ 36 hours later

- Egg collection *

↓ 2, 3, or 5 days later

- Embryos put back into the womb

↓ 2 weeks later

- Pregnancy test
- *Starting on the day of egg collection, you will take a daily dose of Progesterone (to prepare the lining of the womb), which is taken in the form of pessaries inserted into the vagina. These should be taken daily up until the pregnancy test. If the test is positive, the pessaries should be continued until the 8^th^ week of pregnancy.
- *The approximate duration of this treatment regimen (ie from the start of Nafarelin*

*nasal spray, until the pregnancy test is performed) is 4 weeks*.

**3. The GnRH antagonist (Cetrorelix) regimen**

- Day 2/3 of the menstrual cycle: Ovarian stimulation with FSH injections (continued until HCG injection)

↓ approx 6-8 days later (when the largest ovarian follicle has a diameter of 14mm)

- Cetrorelix injections (continued until HCG injection)

↓ approx 9-14 days from the start of FSH

- HCG injection (to mature the eggs)

↓ 36 hours later

- Egg collection *

↓ 2, 3, or 5 days later

- Embryos put back into the womb

↓ 2 weeks later

- Pregnancy test
- *Starting on the day of egg collection, you will take a daily dose of Progesterone (to prepare the lining of the womb), which is taken in the form of pessaries inserted into the vagina. These should be taken daily up until the pregnancy test. If the test is positive, the pessaries should be continued until the 8^th^ week of pregnancy.
- *The approximate duration of this treatment regimen (ie from the start of FSH*

*Injections until the pregnancy test is performed) is 4 weeks.*

**Why have I been chosen?**

You have been given this study information as we have identified from your notes that you fit the criteria (in terms of your ovarian response in your previous treatment cycle) which would make you eligible to take part.

**Do I have to take part?**

No. It is up to you to decide whether or not to take part. If you wish to take part, you will be given this information sheet to keep and will be asked to sign a consent form. You are still free to withdraw your consent at any time and without giving a reason. A decision to withdraw at any time, or a decision not to take part, will not affect the standard of care you receive.

**What will happen if I take part?**

All participants will be randomly allocated to one of the three treatment regimens listed on page 1. You will only participate in one of the treatment regimens. After you have been allocated to a treatment regimen you will follow this throughout your IVF cycle. Each treatment regimen will involve slightly different timelines and different drugs used to suppress your hormones. Once you have been allocated, the doctors and nurses will tell you exactly what you are to do in terms of taking your drugs and clinic visits/ scans etc.

**Expenses and payments:**

The GnRH agonist **(Nafarelin)** and the GnRH antagonist **(Cetrorelix)** will be provided to you free of charge as they are study medications. All other medications (the FSH injections, the HCG injection and the progesterone pessaries) will need to be paid for (either by your Primary Care Trust, your GP or by yourselves).

**What do I have to do?**

You should take your mediations regularly as instructed by our nurses/doctors and fill in your treatment cards accordingly.

**What are the side effects of any treatment received when taking part?**

If you are on the GnRH agonist long regime you might experience some side effects such as hot flushes, night sweats, headaches or mood swings during the two weeks that you take the Nafarelin only. You may not experience all or any of these symptoms but, if you do, they will improve once you start your daily injections to stimulate your ovaries.

**What are the other possible disadvantages and risks of taking part?**

There are no disadvantages or risks of taking part in the study. Taking part in the research will not involve any extra tests or additional visits to the hospital.

**What are the possible benefits of taking part?**

We cannot promise the study will help you personally in this treatment cycle but the information we get might help improve the future treatment of women who had poor ovarian response in a previous IVF treatment cycle.

**What if there is a problem?**

If you have a complaint about the way you have been dealt with during the study or any other matter, you can lodge a complaint. There is more detailed information in Part 2 of this leaflet.

**Will my taking part in this study be kept confidential?**

Yes. All the information about your participation in this study will be kept confidential. Details about this are included in Part 2 of this leaflet.

**Contact Details:**

You should contact Dr Sunkara/ Mr Khalaf at the Assisted Conception Unit on 0207 188 0496 or the address above for any further information or questions about this study. If you need to speak to a doctor outside routine working hours in case of an emergency, you should ring the Unit on 0207 188 2300 and you will be given the number for the on-call doctor from the Unit.

**This completes part 1 of the information sheet.**

**If the information in Part 1 has interested you and you are considering participating, please read the additional information in Part 2 before making any decision.**

**Part 2**

**What if relevant new information becomes available?**

If this happens, Dr Sunkara will tell you about it and discuss with you whether you want to or should continue in the study. If you decide not to carry on, Dr Sunkara will make arrangements for your care to continue. If you decide to continue in the study you will be asked to sign an updated consent form.

**What will happen if I do not want to carry on with the study?**

It is entirely up to you whether or not you wish to take part. If you decide to take part and then change your mind you are free to withdraw at any time without giving a reason and your treatment/care will not be affected in any way.

**What if there is a problem?**

**Complaints**

Any complaint about the way you have been dealt with during the study will be addressed. If you have a concern about any aspect of this study, you should ask to speak to Dr Sunkara or Mr Khalaf who will try their best to answer your questions (contact number 020 718 8046 or at the above address). If you remain unhappy and wish to complain formally, you can do this through the NHS complaints procedure. You can contact the Patients Advisory and Liaison Service (PALS) at Guy’s and St Thomas’ NHS Foundation Trust or you can write to Sir Jonathan Michael, Chief Executive, Guy’s and St Thomas’ NHS Foundation trust, Guy’s Hospital, St Thomas Street, London, SE1 9RT.

**Harm**

Should you be harmed in any way by this study, unfortunately we cannot provide special compensation arrangements. If you are harmed and this is due to someone’s negligence then you may have grounds for a legal action for compensation against Kings College London and Guys and St Thomas’ NHS Foundation Trust, but you may have to pay your legal costs. The normal NHS complaints mechanisms will still be available to you.

**Will my taking part in this study be kept confidential?**

All information collected from you for the purposes of this study will be kept strictly confidential. Any information used outside the hospital will have any identifying details removed so that your data remains completely anonymous. You will not be identified in any publication of results from this study. We will inform your GP of your participation in the trial if you agree.

**Involvement of your General Practitioner (GP)**

We will inform your GP of your participation in the trial if you agree.

**What will happen to the results of the research study?**

We hope to publish the results and information we obtain from this study in a medical journal/s. We will also make the information available on our website for the general public.

**Who is organising and funding the research?**

The study is being organised and funded by the Assisted Conception Unit, Guys and St Thomas’ NHS Foundation Trust.

**Who has reviewed the study?**

This study was given a favourable ethical opinion for conduct in the NHS by St Mary’s Hospital, London Research Ethics Committee.

**Further information:**

Consumers for Ethics in Research (CERES) [www.ceres.org.uk](http://www.ceres.org.uk/) publishes a leaflet entitled ‘Medical Research and You’. This leaflet gives more information about medical research and looks at some questions you may want to ask. A copy may be obtained from: CERES, PO Box 1365, London N16 0BW.

**You will be given a copy of the information sheet and a signed consent form to keep.**

Thank you for taking time to read this sheet and for considering taking part in the study.
