# Supplementary material for: A three-arm randomised controlled trial comparing Gonadotrophin Releasing Hormone (GnRH) agonist long regimen versus GnRH agonist short regimen versus GnRH antagonist regimen in women with a history of poor ovarian response undergoing in vitro fertilisation (IVF) treatment: Poor responders intervention trial (PRINT)
Source: Reprod Health. 2007 Dec 28;4:12. doi: 10.1186/1742-4755-4-12 (PMC2259311; doi:10.1186/1742-4755-4-12)
Supplement: Additional file 2 — Poor Responders Intervention Trial – Consent form. The data provided represents the consent form that is signed by eligible women wishing to participate in the trial. [file 1742-4755-4-12-S2.doc]

**Centre Number:**  0102

**Study Number:** EudraCT No’ 2006- 004460-31

**Name of Researchers:** Mr Y Khalaf and Dr Sunkara

**Patient Identification Number for this trial:**

**CONSENT FORM**

**Title of Project: Poor Responders Intervention Trial (PRINT)**

1. I confirm that I have read and understand the information sheet for this study:

***(“Poor Responders Intervention Trial” Version 1, 20^th^ October 2006)***

I have had the opportunity to consider the information, ask questions and have these answered satisfactorily.

1. I understand that my participation is voluntary and that I am free to withdraw at any time, without giving any reason and without my medical care or legal rights being affected.
2. I give permission for responsible individuals from the Guy’s Assisted Conception Unit to have access to my medical notes (where it is relevant to my taking part in the research).
3. I agree to my GP being informed of my participation in the study .
4. I agree to take part in the above study.

Name of Patient Date Signature

Name of Person taking consent Date Signature

(if different from researcher)

Researcher Date Signature

| When completed: |  |
| --- | --- |
| - 1 form for participant |  |
| - 1 for researcher site file |  |
| - 1 (original) to be kept in medical notes. |  |
