# Supplementary material for: A three-arm randomised controlled trial comparing Gonadotrophin Releasing Hormone (GnRH) agonist long regimen versus GnRH agonist short regimen versus GnRH antagonist regimen in women with a history of poor ovarian response undergoing in vitro fertilisation (IVF) treatment: Poor responders intervention trial (PRINT)
Source: Reprod Health. 2007 Dec 28;4:12. doi: 10.1186/1742-4755-4-12 (PMC2259311; doi:10.1186/1742-4755-4-12)
Supplement: Additional file 3 — Trial case report form. The data provided represents the form in which data from all participating women are recorded. [file 1742-4755-4-12-S3.doc]

Patient Identification Details:

1.	Patient Name (1st name & surname in CAPs)	
2.	Date of Birth	______/______/______     (DD-MMM-YYYY)        Age on day of randomisation:
3.	Subject Number	
4.	Patient IVF Unit Number	
5.	Randomised Intervention	Long Agonist   /    Short Agonist    /    Antagonist
6.	Date subject signed written consent form	______/______/______    (DD-MMM-YYYY)


Name of the person recording the data:	
Signature:	
Date:	_____/______/______       (DD-MMM-YYYY)


Screening Data:

Subfertility:	Type     Male:                    Primary   Secondary       Female:                Primary   Secondary       Couple               Duration:           Cause: Tubal Disease            Ovulatory Disorder            Male Factor             Unexplained            Other:


BMI		Estradiol level	pmol/L
FSH level	iu/l	Antral Follicle Count  (if available)	
		
		


1.	No of previous IVF treatment cycles	
2.	No of previous cancelled treatment cycles due to poor response	
3.	No of previous IVF cycles with </= 3 eggs in the last 5 years	
4.	No of IVF cycles with > 3 eggs in the last 5 years	
5.	No of previous pregnancies	
6.	No of IVF pregnancies	
7.	Total number of live births	


Name of the person recording the data:	
Signature:	
Date:	_____/______/______  (DD-MMM-YYYY)


Stimulation Details:

1.	Age on Day 1 of Stimulation	Yrs
2.	Stimulation Drug, FSH (Dose 450 IU only)	Gonal-F                   Menopur                         Puregon
3.	Number of days of Ovarian Stimulation	
4.	Total dose of FSH	iu/l
5.	HCG medication	Ovitrelle   Dose:                  ius     Pregnyl   Dose:    ius
		


	
	
Name of the person recording the data:	
Signature:	
Date:	_____/______/______  (DD-MMM-YYYY)


Egg Collection:

Egg Collection                                            Yes                                        No      (If No, the form is complete)
1.	Number of follicles >14mm at egg collection	
2.	Number of eggs collected	
3.	Number of mature eggs if ICSI	
4.	Treatment type	IVF                                      ICSI
5.	Number of pronuclei cells	


Name of the person recording the data:	
Signature:	
Date:	_____/______/______  (DD-MMM-YYYY)


Embryo Transfer:

Embryo Transfer                                        Yes  No      (If No, the form is complete)
1.	Day of embryo transfer	2 3 4 5
2.	Number of embryos available for transfer	
3.	Number of embryos transferred	1 2 3
4.	Cumulative embryo score	
5.	Blastocyst quality	
6.	Embryos frozen	Yes                                   No
7.	Day embryos were frozen	1 2 3 4 5
4	Number of embryos frozen	


Name of the person recording the data:	
Signature:	
Date:	_____/______/______  (DD-MMM-YYYY)


Pregnancy Test Result:

Pregnancy Test  Result (Urine or Serum)	Positive                   Negative


Name of the person recording the data:	
Signature:	
Date:	_____/______/______  (DD-MMM-YYYY)


Outcome:

Pregnancy Ultrasound Scan
1.	Number of fetal sacs	1 2 3
2.	Number of fetal poles	1 2 3
3.	Number of fetal hearts	1 2 3


4.	Ongoing Pregnancy	Yes     No
5.	Miscarriage	Yes    No
6.	Ectopic Pregnancy	Yes    No
7.	Biochemical Pregnancy	Yes    No
8.	Other  (Please specify)	Yes    No


Name of the person recording the data:	
Signature:	
Date:	_____/______/______  (DD-MMM-YYYY)


	
OtherOther Other Other
